# Night‐Time Apomorphine Infusion: Who Are the Best Candidates?

**DOI:** 10.1002/mdc3.13799

**Published:** 2023-06-13

**Authors:** Valérie Cochen De Cock, Pauline Dodet, Smaranda Leu‐Semenescu, Cécile Aerts, Beatriz Abril, Giovanni Castelnovo, Nicolas Landragin, Sophie Drapier, Hélène Olivet, Anne‐Gaëlle Corbillé, Laurène Leclair‐Visonneau, Mathieu Anheim, Marie Vidailhet, Isabelle Arnulf, Mohamed Doulazmi, Emmanuel Roze, Post‐hoc analysis of the APOMORPHEE study

**Affiliations:** ^1^ Sleep and Neurology Department Beau Soleil Clinic Montpellier France; ^2^ EuroMov Digital Health in Motion Univ Montpellier, IMT Mines Alès Montpellier France; ^3^ Sleep Disorders Unit Pitié‐Salpêtrière University Hospital Paris France; ^4^ Sleep Department University Hospital of Nîmes Nîmes France; ^5^ Department of Neurology University Hospital of Nîmes Nîmes France; ^6^ Clinique du Millénaire Montpellier France; ^7^ Department of Neurology Pontchaillou INSERM CIC1414 Rennes France; ^8^ Sleep Department Polyclinique Rennes Saint‐Laurent Rennes France; ^9^ Neurology Department University Hospital of Nantes, INSERM, CIC 1413 Nantes France; ^10^ Clinical Neurophysiology Department University Hospital of Nantes, INSERM U1235, Nantes University Nantes France; ^11^ Department of Neurology University Hospital of Strasbourg Strasbourg France; ^12^ Genetic Institute and Molecular and Cellular Biology (IGBMC), INSERM‐U964/CNRS‐UMR7104/ Strasbourg University Illkirch France; ^13^ AP‐HP, Salpetriere Hospital, DMU Neuroscience 6 Paris France; ^14^ Sorbonne University, Paris Brain Institute, Inserm, CNRS Paris France; ^15^ Adaptation Biologique et Vieillissement (UMR8256), Institut de Biologie Paris Seine, Sorbonne University, CNRS Paris France

**Keywords:** Parkinson's disease, motor fluctuations, treatment, sleep, insomnia, sleepiness, apomorphine

## Abstract

**Background:**

We recently demonstrated in a randomized controlled trial (APOMORPHEE, NCT02940912) that night‐time only subcutaneous apomorphine infusion improves sleep disturbances and insomnia in patients with advanced Parkinson's disease and moderate to severe insomnia.

**Objectives:**

To identify the best candidates for receiving night‐time only subcutaneous apomorphine infusion in routine care.

**Methods:**

In this post‐hoc analysis of APOMORPHEE, we compared the characteristics of patients according to whether they chose to continue night‐time only subcutaneous apomorphine infusion at the end of the study period or not.

**Results:**

Half of the patients (22/42) chose to continue the treatment. Off duration (day or night), painful Off dystonia, and insomnia severity at baseline were associated with night‐time only apomorphine continuation. Multivariate analysis retained only Off duration as an independent predictor of continuation.

**Conclusions:**

The best candidates for night‐time only apomorphine are patients with severe and prolonged Off periods (day or night) and severe insomnia.

Insomnia is the most frequent sleep complaint in patients with Parkinson's disease (PD).^1^ Treating this disorder is crucial for patients’ quality of life and their ability to cope with daytime activities.^2^ One interesting emerging hypothesis is that sleep‐related glymphatic clearance might influence disease progression, which further encourage the implementation of treatments for insomnia.^3^, ^4^ Apomorphine is a potent non‐selective dopamine agonist with a pharmacological profile resembling that of levodopa, and with a similar anti‐parkinsonian effect.^5^ It can be clearly differentiated from other commonly used dopamine agonists on the basis of its pharmacology. For example, whereas the action of ropinirole or pramipexole is limited to D2‐like receptors (D2 and D3), apomorphine acts on D1 and D2 receptor classes, including all major subtypes (D1, D2, D3, D4, D5) and has affinity for various serotoninergic and alpha adrenergic receptor.^6^, ^7^ It can be administered subcutaneously from an infusion pump to treat manifestations of advanced PD which are not sufficiently controlled by oral treatment.^8^ Uncontrolled studies have suggested that apomorphine infusion is effective to treat both motor^9^, ^10^, ^11^, ^12^ and non‐motor manifestations in patients with advanced PD,^13^, ^14^, ^15^, ^16^ with sustained benefits over time.^17^, ^18^, ^19^ This efficacy was formally demonstrated in a randomized controlled study over a 12 week‐period using apomorphine infusion during waking hours.^20^

Preliminary pilot studies have raised an interest for the use of night‐time only apomorphine^21^, ^22^ or in addition to its use during daytime.^23^, ^24^

We recently demonstrated in a randomized controlled trial (APOMORPHEE) that night‐time only subcutaneous apomorphine infusion (mean flow 4 mg/h) improved sleep disturbances and insomnia in patients with fluctuating PD and moderate to severe insomnia.^25^ The aim of the current post‐hoc analysis of the APOMORPHEE study was to identify the best candidates for receiving this treatment in routine care based on their choice to continue the treatment at the end of the study or not.

## Methods

### Patients and Study Design

APOMORPHEE was a randomized, double‐blind, placebo‐controlled, multicenter crossover study. It enrolled patients with fluctuating PD who complained of moderate to severe insomnia (Insomnia Severity Index [ISI] > 15). The full study design has been previously published.^25^ Briefly, the study comprised two treatment periods during which patients were treated night‐time only. The treatment periods were separated by a 14‐night washout period. Each patient was randomly allocated to one of the treatment sequences (apomorphine followed by placebo, or vice versa). Each treatment period was divided into three phases: (i) a 10‐night titration phase, (ii) a 7‐night maintenance phase and, (iii) a 3‐night de‐escalation phase. Participants who completed the two periods of the study were then asked whether they wanted to continue with apomorphine treatment or not. Of note, baseline treatments were kept stable all study long.

The study protocol was conducted in accordance with Good Clinical Practice and the declaration of Helsinki. It was registered with ClinicalTrials.gov, NCT02940912 and approved by a national ethics committee (CPP Sud Méditerranée III, Nîmes, France, ID‐RCB 2015–005793‐37). Written informed consent was obtained from all participants.

### Outcome Measures

The primary outcome measure of APOMORPHEE was the mean change in sleep quality as evaluated by Parkinson's Disease Sleep Scale (PDSS). Secondary outcomes evaluated the change in various motor and non‐motor manifestations of PD. In this post‐hoc analysis, our aim was to identify factors that could have influenced the decision of the patients to continue apomorphine, by comparing the baseline characteristics of the patients who decided to continue with apomorphine and those who preferred not to. The baseline outcome measures that were compared included (i) demographics; (ii) disease characteristics namely age at onset, disease duration, non‐motor and motor aspects of daily living, On‐state motor examination and motor complications respectively assessed by part I, II, III and IV of the Movement Disorders Society Unified Parkinson's Disease rating scale (MDS‐UPDRS), and cognitive examination using the Montreal Cognitive Assessment (MoCA); (iii) sleep characteristics, namely PDSS, using the Insomnia Severity Index (ISI) and the presence of clinical rapid eye movements sleep behavior disorder (RBD); and, (iv) treatments.

### Statistical Analyses

Statistical analysis was conducted with the R software (4.0.2) system. The effect of treatment (Apomorphine or Placebo) and of the patient groups (discontinuation or continuation) were analyzed using the general linear model. Treatment and effect of the patient group were assumed as fixed effects and subject‐within‐sequence was treated as a random effect in the model. A multivariate logistic regression model was performed to further explore the determinants of apomorphine continuation.

We performed group analyses of the baseline characteristics comparing the two patient groups (discontinuation or continuation). Variables that failed the Shapiro–Wilk or the Levene tests were analyzed with nonparametric statistics. Categorical variables were compared using Pearson's χ^2^ test or Fisher's exact test. Quantitative variables were analyzed using the Student's *t*‐test or Mann–Whitney's test. Variables were selected to be included in the model either based on the statistical significance on group analysis, or because we thought they might be important to explain apomorphine continuation. We also avoided redundancy between the variables included in the model. The multivariable regression model was based on the stepwise method. The data are expressed as mean (SD), and *P* < 0.05 was assumed to be statistically significant.

## Results

Of the 46 patients randomized in APOMORPHEE, 42 completed the two study periods and were thus included in the post‐hoc analysis. Among them, 22 decided to continue with apomorphine and 20 preferred not to. Comparison of the baseline characteristics between the two groups are reported in Table 1. Compared to those who stopped, patients who continued apomorphine had longer Off periods (day or night) (*P* = 0.04) and a higher proportion of Off periods spent with painful dystonia (*P* = 0.01). They also had more severe insomnia (*P* = 0.02) and sleep complaints (*P* = 0.0144). Of note, they did not differ for sleepiness (*P* = 0.3644).

**TABLE 1:** Comparisons of the characteristics of the patients who decided to continue or withdraw subcutaneous night‐time only apomorphine infusion at the end of the APOMORPHEE study

Clinical characteristics	Total	Apomorphine		*P* value
		Withdrawal	Continuation	
Number of subjects	42	20	22	
Sex, Male	25 (60%)	10 (50%)	15 (68%)	0.23
Age (in years)	64.0 (9.3)	65.29 (7.76)	62.76 (12.37)	0.45
BMI (kg/m^2^)	26.1 (5.6)	26.73 (7.20)	25.54 (3.87)	0.89
Disease characteristics				
Disease duration (years)	10.0 (4.6)	10.30 (5.56)	9.72 (3.57)	0.70
Age at onset	53.9 (10.6)	55.0 (9.08)	52.95 (11.97)	0.92
MDS‐UPDRS Part I	15.9 (6.5)	15.80 (6.30)	15.95 (6.79)	0.99
MDS‐UPDRS Part II	16.4 (7.8)	15.35 (6.16)	17.41 (9.04)	0.39
MDS‐UPDRS Part III score during on	26.0 (15.4)	22.85 (14.16)	28.86 (16.28)	0.23
MDS‐UPDRS Part IV	6.3 (3.8)	5.00 (3.24)	7.50 (3.94)	0.16
Part IV‐1 Dyskinesia duration	0.57 (0.73)	0.5 (0.61)	0.64 (0.85)	0.79
Part IV‐2 Consequences of dyskinesia	0.45 (0.99)	0.2 (0.41)	0.68 (1.29)	0.42
Part IV‐3 Off periods duration	1.17 (0.66)	0.95 (0.60)	1.36 (0.66)	**0.04**
Part IV‐4 Off periods consequences	1.88 (1.25)	1.6 (1.35)	2.14 (1.13)	0.15
Part IV‐5 Motor fluctuations complexity	1.19 (0.97)	1.05 (0.94)	1.32 (0.99)	0.33
Part IV‐6 Painful off dystonia	1.05 (1.03)	0.7 (0.92)	1.36 (1.05)	**0.01**
MoCA	27.6 (1.9)	27.75 (0.68)	27.41 (2.11)	0.56
Sleep characteristics				
PDSS (/150)	80.0 (17)	86.68 (12.51)	73.95 (22.05)	0.014
Item‐4 Nocturnal restlessness	6.2 (3.23)	6.81 (2.89)	5.66 (3.48)	0.66
Item‐8 Nocturia	2.6 (3.04)	2.77 (3.25)	2.45 (2.89)	0.94
Item15 Daytime dozing	7.9 (2.72)	7.71 (2.65)	8.08 (2.85)	0.36
Insomnia severity index (ISI)	19.4 (3.6)	18.00 (3.51)	20.60 (3.40)	**0.02**
Clinical RBD	18 (43.9%)	9 (47%)	9 (41%)	0.68
Treatments				
Antiparkinsonian medication				
Daily levodopa‐equivalent dose	741.3 (313.2)	700.00 (287.73)	778.79 (336.88)	0.74
Dopamine agonist	29 (69%)	14 (70%)	15 (68%)	0.90
Prolonged release	26 (62%)	13 (65%)	13 (59%)	0.9
Immediate release	3 (7%)	1 (5%)	2 (9.1%)	1
Equivalent daily dose	134.9 (128.5)	115.6 (109.8)	152.5 (143.7)	0.5

*Note*: Data are *n* (%) or mean (SD). In bold: *P* < 0.05.

Abbreviations: COMT, Catechol‐O‐methyltransferase; MAOB, monoamine oxidase type B; MDS‐UPDRS, Movement Disorder Society Unified Parkinson's Disease Rating Scale; MoCA, Montreal Cognitive Assessment.

The independence of factors predicting the decision to stop or continue apomorphine was assessed by stepwise multivariate logistic regression with the decision to continue apomorphine as a response variable, and severity of insomnia, Off duration, painful Off dystonia, age, nocturnal restlessness, nocturia, and daytime dozing as predictive variables (Table 2). From this model (Adjusted *R*2 = 0.337), only Off duration (day or night) was found to independently predict the decision to continue apomorphine (OR = 3.859, 95% CI 1.011–14.724, *P* = 0.048): the greater the Off duration, the more likely it was that the patient would decide to continue apomorphine treatment. Severity of insomnia also tended to predict independently continuation of apomorphine (OR = 0.783, 95% CI 0.976–1.574, *P* = 0.078).

**TABLE 2:** Factors related predicting the continuation of the apomorphine treatment at the end of APOMORPHEE study

Factors predicting apomorphine continuation	*β*	S.E	*P*‐value	OR (95% CI)
Insomnia severity index (ISI)	0.215	0.122	0.078	1.239 (0.976–1.574)
Off periods duration (MDS‐UPDRS Part IV‐3)	1.35	0.683	0.048*	3.859 (1.011–14.724)
Daytime dozing (Item15 of PDSS)	−0.244	0.155	0.115	0.783 (0.578–1.061)	

*Note*: Multivariate logistic regression analysis using the forward stepwise selection method; with decision to continue apomorphine as a response variable and severity of insomnia, Off periods duration, painful Off dystonia, age, nocturnal restlessness, nocturia, daytime dozing as predictive variables. *β*: partial regression coefficient; OR (EXP [*β*]). Odds Ratio value of the corresponding variable with 95% Confidence Interval. In bold: *P* < 0.05.

Abbreviation: MDS‐UPDRS, Movement Disorder Society Unified Parkinson's Disease Rating Scale.

## Discussion

Off duration, painful Off dystonia, and insomnia severity at baseline were associated with continuation of night‐time apomorphine. Multivariate analysis retained Off duration alone as an independent predictor of continuation. Baseline sleepiness was not a predictor of discontinuation.

The main strength of our work is that we analyzed data from a randomized, controlled study.^25^ Its originality lies in our focus on the patient's decision to continue the treatment or not as a way of identifying the best candidates for this treatment in clinical routine in line with a participatory approach to personalized medicine. This decision takes into account efficacy, tolerability, and acceptability of the infusion pump.

The limitations include, first, the post‐hoc analysis, on a small group of patients, second the relatively short 1 week duration at the targeted dose of apomorphine which probably missed longer‐term effects, and third, that we evaluated the continuation or discontinuation of night‐time apomorphine just at the end of the study period thereby possibly misclassifying some patients who may have decided to stop treatment later, or others who may have come back on their decision to discontinue the treatment.

Treatment with apomorphine infusion specifically targets Off periods that are no longer controlled by oral treatments in patients with PD.^11^, ^20^, ^26^ The beneficial effect is sustained over time.^18^, ^19^ This is consistent with our findings that 24 hr Off duration and severity are associated with the decision to continue night‐time only apomorphine. When Off periods extend to night‐time, they can be particularly distressing for patients who lie awake at night and who are unable to move in bed, empty their bladder, or change their bedding, and who may suffer from painful dystonia or rest tremor. Apomorphine, a pro‐sleep and pro‐motor drug, is understandably appreciated by affected patients as it can alleviate this difficult time either by inducing a better sleep pattern or by improving mobility and enabling the patient to get to the bathroom. Off duration and severity could be considered as markers of continuous dopamine deficiency–the greater the duration and severity, the greater the dopamine deficiency–which is by definition not limited to daytime. Together this suggests that compensation of nocturnal dopamine deficiency with night‐time apomorphine infusion provides critical benefits on sleep quality in patients with fluctuating PD, further supporting the concept of 24 hr continuous dopamine stimulation. Interestingly, 13 of the 22 patients continuing apomorphine after the study were already treated with prolonged release dopamine agonists at baseline (kept stable all study long). This suggests that the continuous dopaminergic stimulation was insufficient or not sustained enough, and that the patients benefited from higher doses and more continuous treatment. Alternatively, the additional effect of apomorphine on sleep beyond that of oral/transdermal dopamine agonists could rather be related to its broad spectrum dopaminergic action on all dopamine D1‐like and D2‐like receptors and/or to its off target action on serotonin and alpha adrenergic receptors.^6^, ^7^

Continuous apomorphine infusion is a device‐aided therapy for fluctuating PD involving some practical constraints in daily life. However, it is minimally invasive, reversible, and easy to use. More than half of the patients of our study (22/42) chose to continue the treatment indicating not only a satisfactory efficacy/tolerability ratio but also good acceptability. Night‐time only use may explain this acceptability and opens the perspective for routine use of this treatment strategy. The best candidates for this treatment are patients with severe and prolonged Off periods (day or night) and severe insomnia. In some patients with fluctuating PD, relief of insomnia can require significant dopaminergic stimulation during night‐time, which can be effectively achieved with night‐time only apomorphine infusion.

## Author Roles

(1) Research Project: A. Conception, B. Organization, C. Execution; (2) Statistical Analysis: A. Design, B. Execution, C. Review and Critique; (3) Manuscript Preparation: A. Writing of the first draft, B. Review and Critique.

V.C.D.C.: 1A, 1B, 1C, 2A, 2C, 3A, 3B

E.R.: 1A, 1B, 1C, 2A, 2C, 3A, 3B

P.D.: 1C, 3C

S.L.: 1C, 3C

C.A.: 1C, 3C

B.A.: 1C, 3C

G.C.: 1C, 3C

N.L.: 1C, 3C

S.D.: 1C, 3C

H.O.: 1C, 3C

A.G.C.: 1C, 3C

L.L.V.: 1C, 3C

M.A.: 1C, 3C

I.A.: 3B

M.A.: 3B

M.V.: 3B

M.D.: 2A, 2B, 2C

All authors read and approved the final manuscript.

## Disclosures

**Ethical Compliance Statement:** The study protocol was conducted in accordance with Good Clinical Practice and the declaration of Helsinki. It was approved by a national ethics committee (CPP Sud Méditerranée III, Nîmes, France, ID‐RCB 2015–005793‐37). Written informed consent was obtained from all participants. We confirm that we have read the Journal's position on issues involved in ethical publication and affirm that this work is consistent with those guidelines.

**Funding Sources and Conflicts of Interest:** This was an academic, investigator‐initiated study with funds from Orkyn Ltd and Aguettant Pharma, Société Française de Médecine Esthétique, Enjoysharing. All authors declare no conflict of interest concerning the research related to the manuscript.

**Financial Disclosure for the Previous 12 Months:** VCDC received travel grants from ORKYN. ER received honorarium for speech from Orkyn, Aguettant, Elivie and for participating in an advisory board from Merz‐Pharma. He received research support from Merz‐Pharma, Orkyn, Aguettant, Elivie, Ipsen, Everpharma, Enjoysharing, Fondation Desmarest, AMADYS, ADCY5.org, Fonds de dotation Patrick Brou de Laurière, Agence Nationale de la Recherche, Societé Française de Médecine Esthétique, Dystonia Medical Reasearch Foundation. MA received consultancies honoraria from Abbvie, Luxia Merz, Orkyn, Asdia, Prata, Ipsen. LLV received research partnership (cost of analysis) from Luxia, SL received travel grants from ELIVIE and UCB. HO, BA, NL, PD, AGC, CA, GC, MD, MV, IA have no additional disclosures to report.

## Data Availability

The data that support the findings of this study are available from the corresponding author upon reasonable request.
